# Distributions of Fecal Indicators at Aquaculture Areas in a Bay of Republic of Korea

**DOI:** 10.4014/jmb.2406.06001

**Published:** 2024-10-16

**Authors:** SungJun Park, Cheonghoon Lee, Sung Jae Jang, Kyuseon Cho, Jin Hwi Kim, Woon-Ki Kim, Joo-Hyon Kang, Kwon-Sam Park, GwangPyo Ko

**Affiliations:** 1 Department of Environmental Health Sciences, Graduate School of Public Health, Seoul National University, Seoul 08826, Republic of Korea; 2 N-Bio, Seoul National University, Seoul 08826, Republic of Korea; 3 Institute of Health and Environment, Seoul National University, Seoul 08826, Republic of Korea; 4 Department of Civil and Environmental Engineering, Konkuk University-Seoul, Seoul 05029, Republic of Korea; 5 Department of Civil and Environmental Engineering, Dongguk University, Seoul 04620, Republic of Korea; 6 Department of Food Science and Biotechnology, Kunsan National University, Gunsan 54150, Republic of Korea

**Keywords:** Fecal contamination, fecal indicator, geographical information system, male-specific coliphage, microbial source tracking, somatic coliphage

## Abstract

Aquaculture products, such as clams, scallops, and oysters, are major vectors of fecal-derived pathogens. Male-specific and somatic coliphages are strongly correlated with human noroviruses, the major enteric viruses worldwide. Geographic information system with local land-use patterns can also provide valuable information for tracking sources of fecal-derived pathogens. We examined distributions of four fecal indicator microorganisms, *i.e.*, male-specific and somatic coliphage, total coliform, and *Escherichia coli* (*E. coli*) in three river and seawater sampling sites located on the coast of Gomso Bay in the Republic of Korea during the sampling period (from March 2015 to January 2016). Geospatial analyses of fecal indicators and correlations between environmental parameters and fecal indicators or among fecal indicators were also performed. Overall, river water samples showed highest concentrations of both types of coliphage in summer (July 2015). High concentrations of both total coliform and *E. coli* were detected in river water during the period from July to September 2015. High concentrations of all fecal indicators were found at site GL02, located in the innermost part of Gomso Bay, which has high-density agriculture and residential areas. Environmental factors related to precipitation—cumulative precipitation on and from 3 days before the sampling day (Prep-0 and Prep-3, respectively)—and salinity were strongly correlated with the concentrations of all fecal indicators. The present results suggest that investigations of multiple fecal indicators with systemic geospatial information are necessary for precisely tracking fecal contaminations of aquaculture products.

## Introduction

Fecal-contaminated aquaculture products, such as clams, scallops, and oysters, are important vectors of pathogenic microorganisms. Especially, enteric viruses, pathogenic *Vibrio* spp., and parasite protozoans, including *Cryptosporidium* spp., can accumulate in oyster, which is a major aquaculture product worldwide [1-3]. Oyster is often consumed raw or undercooked, which can lead to extensive outbreaks caused by contaminated oysters [4, 5]. Therefore, tracking fecal contamination, which is a significant source of pathogens [6], in water near aquaculture areas is crucial to prevent waterborne infections and secure the quality of aquaculture products.

Previous studies have suggested various microbial indicators to identify fecal contamination in water. Concentrations of fecal indicator bacteria (FIB), such as total coliforms, fecal coliforms, and *Escherichia coli* (*E. coli*), have been used for monitoring microbiological burden of water [7-9]. FIB detection is an easy and economical method for tracking fecal contamination, however, various limitations, including short survival rates of FIB in water, inability to determine the source of contamination, and vulnerability to external environmental conditions, such as use of disinfectants or change of pH, reduce the reliability of this method [10]. Moreover, bacteria and enteric viruses differ in their characteristics and ecology, therefore, FIB are not suitable for the prediction of viral contaminations in water [10, 11]. Previous studies have suggested that male-specific coliphages show distinct characteristics according to their origin [12, 13]. Male-specific and somatic coliphages were also shown to be strongly correlated with human noroviruses, the major enteric viruses worldwide [13-15]. Applications of multiple microbial indicators are needed for the precise prediction of fecal contamination in aquaculture products.

Geographic information system (GIS) with local land-use patterns can also provide valuable information for tracking sources of fecal contamination [15-17]. Population and land use patterns have been shown to have significant effects on fecal contamination of surrounding areas [18]. Our previous studies suggested that fecal indicator-combined analyses with the systemic GIS can elucidate major sources of fecal contamination in residential areas or agriculture areas, or predict non-point fecal sources of surface water or seawater sites near aquaculture areas properly [15, 17].

In this study, we investigated distributions of male-specific and somatic coliphages, total coliform, and *E. coli* in three rivers and seawater sampling sites surrounding aquaculture sites in Gomso Bay, one of the major aquaculture areas in Republic of Korea, during the sampling period (from March 2015 to January 2016). Furthermore, we predicted potential fecal sources using fecal indicator-combined geospatial analyses and confirmed correlations of fecal indicators with environmental parameters and other fecal indicators in water samples. These observations will facilitate the tracking and prevention of fecal contamination in aquaculture products in Gomso Bay.

## Materials and Methods

### Sampling Sites

**Fig. 1:**
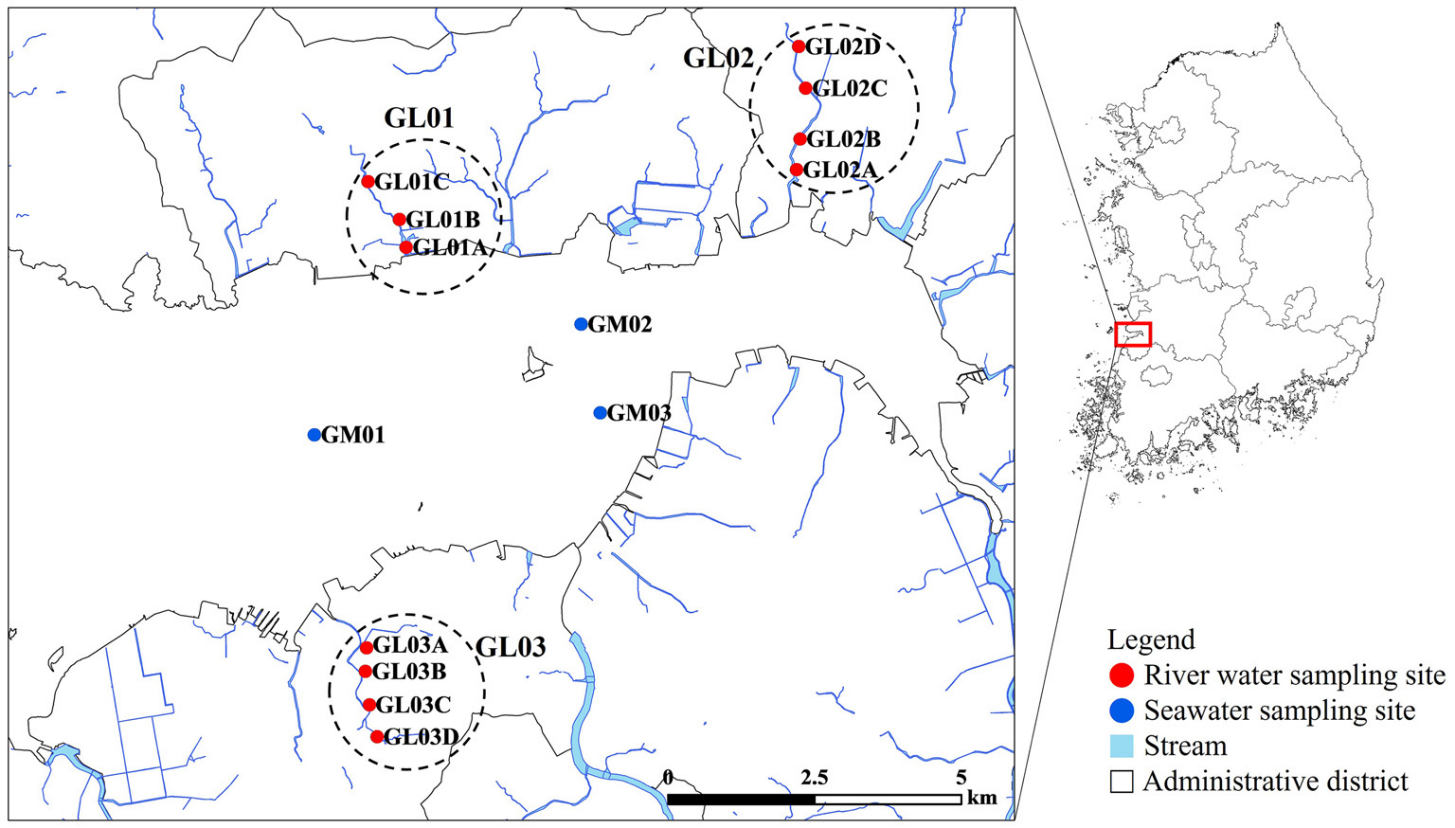
Sampling sites in this study. They were labeled using the initial of the bay (G for Gomso Bay), the water type (M for marine; L for land) with a number indicating the sampling location (1, 2, or 3), and the sampling place (A, B, or C).

We chose three rivers adjacent to the coast of Gomso Bay, Republic of Korea (Fig. 1). Three or four sampling sites located on each river were selected with the considerations of local land-use patterns (*i.e.*, residential, industrial, commercial, and agricultural areas). Three seawater sampling sites were also chosen for tracking distributions of fecal indicators. A total of eighty river water samples were collected from eleven sampling sites between January 2015 and January 2016. Additionally, eight seawater samples were collected from three sampling sites during the summer (July 2015). Environmental parameters were measured for each water sample as described previously [15, 17]. A multiparameter instrument (Professional Plus; Yellow Springs Instruments, USA) was used to measure *in situ* water temperature, pH, conductivity, and turbidity. Other parameters, such as wind speed, relative humidity, air temperature, cumulative precipitation on the sampling day (Prep-0), and a total cumulative precipitation from the 3 days before the sampling day (Prep-3), were obtained from the Korea Meteorological Administration (https://www.kma.go.kr/kma/).

### Enrichment of Coliphage in Water Sample

Water samples were concentrated for coliphage detection as described previously [15, 17]. Briefly, 100 L of each water sample was filtered using NanoCeram powdered activated carbon filter cartridges (Argonide Corp., USA) *in situ*. Then, filters were stored at 4°C until use and eluted three times using a peristaltic pump and the elution buffer containing 3.0% beef extract (BD Biosciences, USA) and 0.1 M glycine (pH 9.5) (Duchefa, USA) for 5 min each time. Eluates were subsequently precipitated under acidic condition (pH 3.5) using 1 M HCl and stirred for 30 min at room temperature. After centrifugation (2,500 ×*g* for 15 min at 4°C), pellets were collected and re-suspended in 0.15 M sodium phosphate (pH 9.0–9.5). After concentration by centrifugation (10,000 ×*g* for 10 min at 4°C), supernatants were adjusted to pH 7.0–7.5. Syringe filters (0.22-μm pore size; Millipore, USA) were used for filtration of supernatants. Final eluents were stored at -80°C until use.

### Quantification of Coliphages in Water Samples

The single agar layer method was used to quantify coliphages in water samples with some modifications [15, 17, 19]. Initially, 0.5 ml of concentrated eluent and 0.3 ml of log-phage host *E. coli* (approximately 0.3 optical density), were co-cultured in 30 ml of tryptic soy broth (BD Biosciences) containing 0.8% agar for 18 h at 37°C in a shaking incubator (150 rpm). *E. coli* F_amp_ (F_amp_; ATCC 700891) and *E. coli* CN13 (CN13, ATCC 700609) were used as hosts for male-specific coliphages and somatic coliphages, respectively. Ampicillin (100 μg/ml; Sigma-Aldrich, USA) and nalidixic acid (100 μg/ml; Sigma-Aldrich) were applied for cultivation of F_amp_ and CN13, respectively. MS2 (ATCC 15597-B1) and PhiX174 (ATCC 13706-B1) coliphages were applied as positive controls of male-specific and somatic coliphages, respectively. Then, plaque-forming units (PFU) in 1 L of water samples were calculated properly.

Isolated plaques were used for coliphage enrichment. Briefly, plaques were suspended in 5 ml of tryptic soy broth (BD Biosciences) containing 100 μl of log-phase host *E. coli* and co-cultured for 18 h at 37°C in a shaking incubator (150 rpm). Subsequently, 5 ml of chloroform was added and the solution was vortexed intensely for 5 min. After centrifugation (5,000 ×*g* for 20 min at 4°C), the purified supernatant with coliphages was stored at -80°C until use.

### Quantification of Total Coliforms and *E. coli*

The standard most probable number (MPN) method was used to quantify total coliforms and *E. coli* with some modifications [20, 21]. Tenfold serial dilutions of water samples were inoculated into five tubes containing lauryl tryptose broth (BD Biosciences) and incubated for 48 h at 35°C. Gas-producing samples were confirmed and cultured in brilliant green bile broth (Oxoid, UK) for 48 h at 35°C or EC-MUG (4-methylumbelliferyl-BD-glucuronide) broth (BD Biosciences) for 24 h at 44.5°C to distinguish total coliforms or *E. coli*, respectively. Confirmation of *E. coli* was performed using an UV-A lamp (365 nm, 6 W). The MPN table was applied to calculate total coliforms and *E. coli* in water samples.

### Fecal Indicator-Combined Geospatial Analyses

Geoprocessing and spatial mapping for fecal indicator-combined analyses were suggested using ArcGIS ver. 10.2.2 (ESRI, USA), as described previously [15, 17]. Data related to Gomso Bay, such as the administrative district, land-use pattern, watershed, and sewage treatment plant, were obtained from the Spatial Big Data Platform provided by the Ministry of Land, Infrastructure and Transport in Republic of Korea (http://geobigdata.go.kr/).

### Statistical Analyses

A GraphPad Prism ver. 10 (GraphPad Software, Inc., USA) was used for statistical analysis and visualization. The limit of detection (LOD) of coliphages and bacterial indicators were 2.1 × 10^-1^ PFU/L and 1.8 × 10^-2^ MPN/100 ml, respectively. Samples below LOD were considered as not detected (ND). Spearman’s rank correlation coefficients among environmental factors and fecal indicators were calculated and visualized using the corrplot (ver. 0.92) package of R Statistical Software (ver. 4.1.2) [22] and GraphPad Prism ver. 10.

## Results

### Environmental Parameters of Sampling Areas

Table 1 shows mean environmental parameters of river water sampling areas during the sampling period. The water temperature exceeded 22°C in July (22.5°C) and September (22.8°C) 2015. In January 2016, Gomso Bay recorded the lowest water temperature (4.5°C). During the entire sampling period, water samples maintained neutral pH within the range of 7.0 to 7.7. In May 2015, Gomso Bay showed the highest conductivity (668.8 μS/cm), salinity (0.4 psu), and turbidity (23.6 NTU). The highest wind speed (6.4 m/s) occurred in March 2015 and the highest relative humidity (91.7%) and air temperature (23.6°C) were recorded in July 2015. In the same month, Gomso Bay showed the highest value of Prep-0 (6.8 mm) and Prep-3 (62.5 mm).

Table 2 shows a summary of averaged environmental parameters of seawater in July 2015. Seawater areas showed significantly higher conductivity (30,757.9 μS/cm) and salinity (30.7 psu) compared to river water areas, which had conductivity of 112.5 μS/cm and salinity of 0.1 psu. In addition, seawater areas showed lower turbidity (8.0 NTU) and wind speed (1.6 m/s) compared to river water areas, which had turbidity of 15.2 NTU and wind speed of 4.1 m/s. There was no precipitation on sampling days for seawater during July 2015.

### Fecal Indicators in River Water Samples

**Fig. 2:**
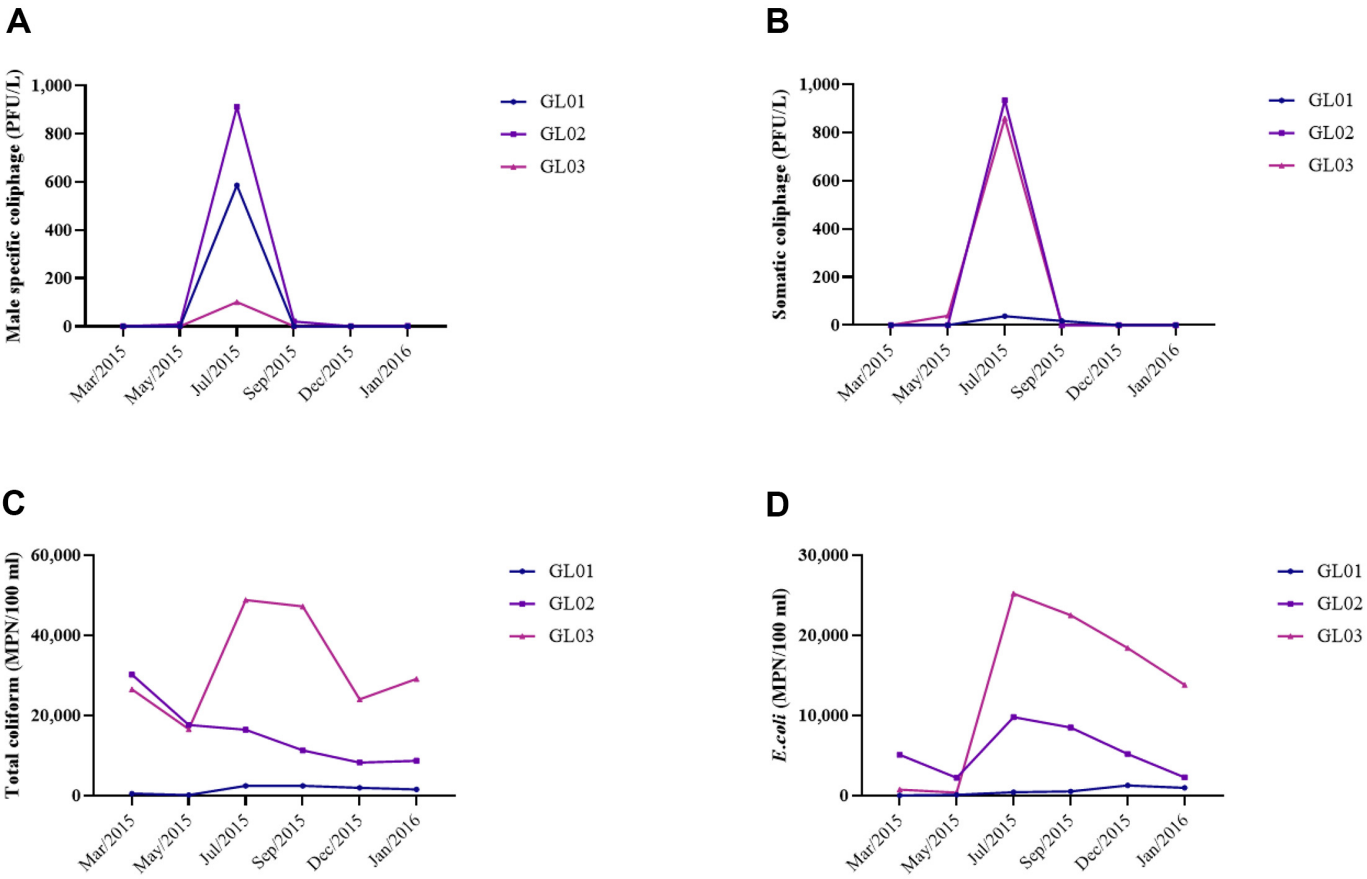
Mean concentrations of various fecal indicators in river water samples. (**A**) Male-specific coliphage; (**B**) Somatic coliphage; (**C**) Total coliform; (**D**) *E. coli*.

Fig. 2 shows mean concentrations of fecal indicators in river water samples. In July 2015, all three river water samples showed the highest concentrations of male-specific and somatic coliphages (Fig. 2A and 2B). Over 800 PFU/L of both types of coliphage were recovered in site GL02 in July 2015. However, in spring (March to May 2015) and winter (December 2015 to January 2016), both types of coliphages were rarely recovered in river water.

During July to September in 2015, site GL03 showed the highest concentrations of total coliforms and *E. coli* among the sampling sites (Fig. 2C and 2D). Remarkably, over 20,000 MPN/100 ml of total coliform were recovered from the water samples of site GL03 in winter. Both site GL02 and GL03 showed clear downward trends in *E. coli* concentrations following the summer season.

### Fecal Indicators-Combined Geospatial Analysis

**Fig. 3:**
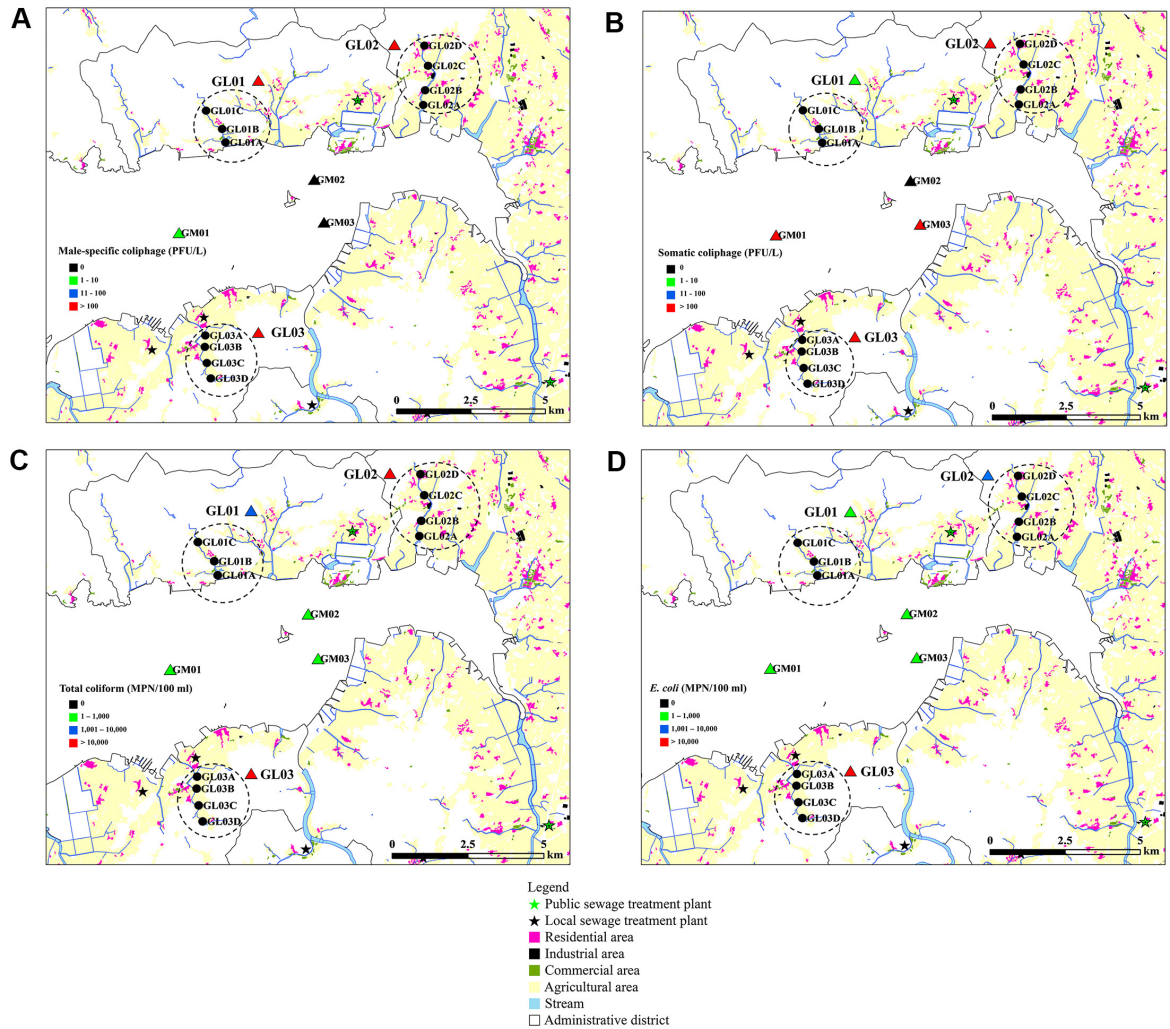
Fecal indicator-combined geospatial map with land-use patterns at sampling sites. (**A**) Male-specific coliphage, (**B**) Somatic coliphage, (**C**) Total coliform, (**D**) *E. coli*.

Results of geospatial analysis with mean concentrations of various fecal indicators are shown in Fig. 3. In all river water sampling areas, male-specific coliphages exceeded 100 PFU/L whereas only site GM01 showed low concentrations of male-specific coliphages (Fig. 3A). Conversely, concentrations of somatic coliphages were high in site GM01 and GM03, which are located in close proximity to site GL03 and GM02, respectively (Fig. 3B). Site GL02, located in the innermost part of Gomso Bay and surrounded by high-density agriculture and residential areas, showed high concentrations of all fecal indicators. Low concentrations of total coliforms and *E. coli* were recovered in all seawater sampling areas (Fig. 3C and 3D).

### Correlations of Fecal Indicators with Environmental Parameters or Other Fecal Indicators in Water Samples

**Fig. 4:**
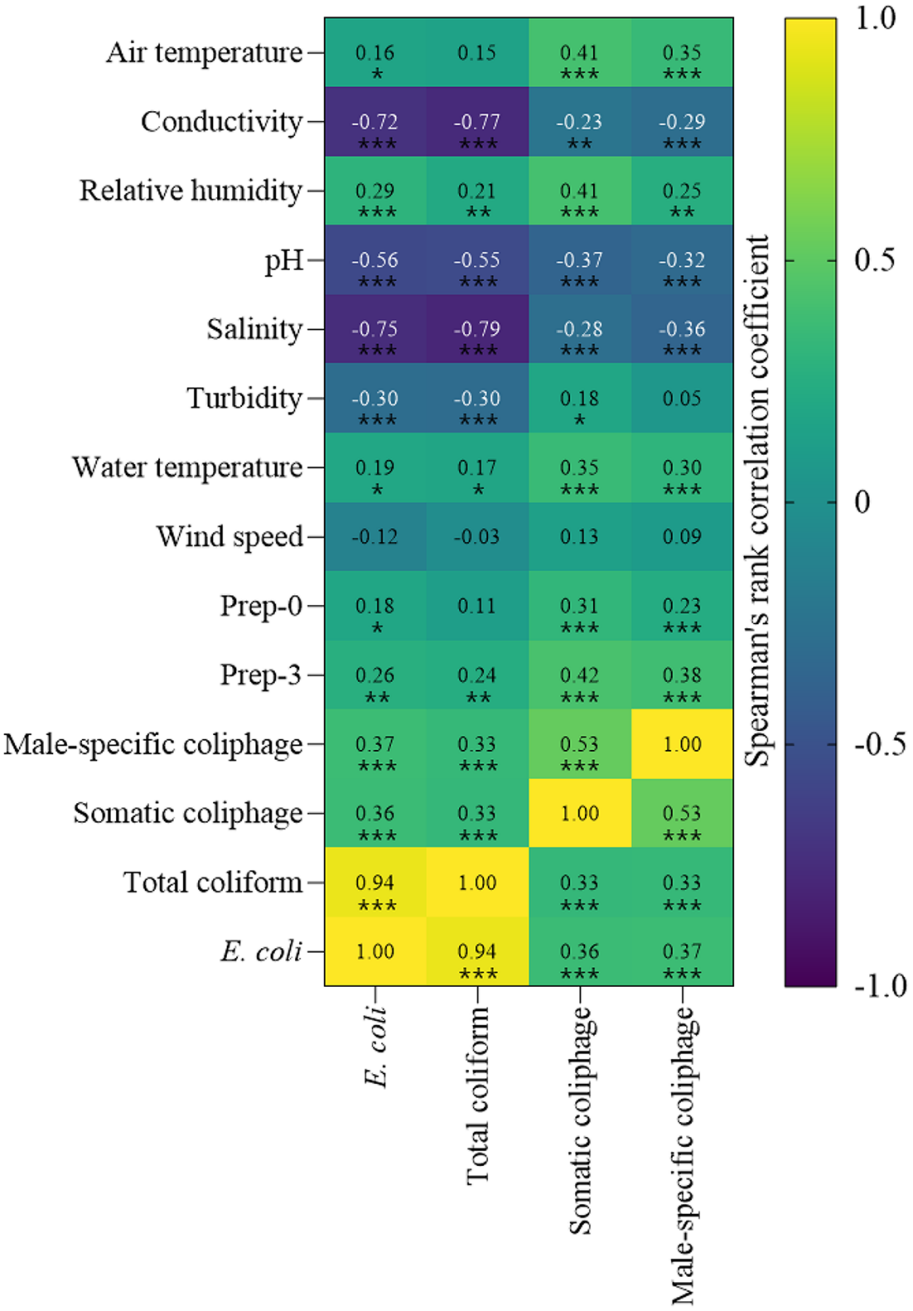
Spearman’s correlation coefficients between environmental parameters and fecal indicators or among fecal indicators in water samples. Asterisks indicate statistical significance (* *p* < 0.05; **p < 0.01; ***, *p* < 0.001).

Fig. 4 exhibits the correlations between environmental parameters and fecal indicators in water samples. Overall, environmental parameters showed strong correlations with concentrations of bacterial indicators compared to coliphages. Conductivity and salinity had clear negative correlations with concentrations of all fecal indicators. Especially, Prep-0 and Prep-3 were positively correlated with *E. coli* and both types of coliphages. In addition, air and water temperature, as well as relative humidity, showed strong correlations with coliphage concentrations.

A significant positive correlation between concentrations of total coliforms and *E. coli* were observed in water samples (Fig. 4). In addition, concentrations of coliphages were positively correlated with concentrations of total coliforms or *E. coli* during the sampling period.

## Discussion

In the present study, we confirmed that river water samples contained high concentrations of fecal indicators compared to seawater samples, consistent with our previous research [17]. Typically, accumulations of fecal contamination occur downstream of the river [23, 24]. Wastewater from residential or agricultural areas is the important source of fecal contamination for river water and can be diluted after release into the sea. Therefore, types and concentrations of fecal indicators are useful for microbial source tracking in aquaculture sites.

Peak concentrations of all fecal indicators were observed during the summer (Fig. 2). Rainfall plays a crucial role in the transportation of feces, leading to elevated concentrations of fecal indicators. This can be attributed to the prolonged rainy season during the summer in the Republic of Korea, which typically extends from mid-June to the end of July [15, 17]. In addition, cumulative precipitation can directly influence the concentrations of fecal indicators and alter salinity or turbidity, which are key environmental parameters affecting the concentrations of viruses and bacteria in water [17, 25].

GIS with concentrations of fecal indicators is a powerful tool for predicting potential sources of fecal contamination. Site GL02 or GL03, which are located close to densely populated residential and agricultural areas, showed the highest concentrations of fecal indicators (Fig. 3). Our results also suggested that site GM01 and GM02 showed higher somatic coliphages than male-specific coliphages (Fig. 3B). Previous studies have suggested that concentrations of both somatic and male-specific coliphages increase during heavy rainfall events [14]. Heavy rainfall can transport various fecal contaminants from terrestrial areas near streams or rivers. However, male-specific coliphages showed greater vulnerability to high temperatures compared to somartic coliphages [26, 27]. Salinity also had negative effects on concentrations and persistances of coliphages [28]. The susceptibility of total coliforms and *E. coli* in high salinity could explain the lower concentrations of these bacterial indicators in seawater sites as compared to river water sites (Fig. 3C and 3D). Furthermore, the aquaculture areas in Gomso Bay are located on the open-type coast, therefore, fecal contaminants from river water cannot be accumulated over long periods in these areas. The treatment levels at sewage treatment plants and the concentrations of fecal indicators they release directly influence the levels of fecal indicators in Gomso Bay. Specially, the local sewage treatment plant at site GL03 could impact the concentrations of fecal indicators significantly at site GM01. Changes in population or land-use patterns, which can vary throughout the entire sampling period, could be utilized to analyze the impact of microbial indicators on tracking fecal contamination [15, 17]. Therefore, it is essential to conduct further longitudinal studies using predictive models that consider seasonal, spatial, and environmental variations. These studies are necessary to understand the distributions of various fecal indicators and their associations with different waterborne pathogens.

Interestingly, this study demonstrated strong positive correlations between concentrations of both types of coliphages, consistent with our previous findings (Fig. 4) [15]. Additionally, coliphage concentrations showed strong correlations with various environmental parameters, such as air and water temperature, and relative humidity (Fig. 4) [15]. Concentrations of bacterial indicators, total coliforms and *E. coli*, exhibited clear negative correlations with water quality parameters, such as conductivity and salinity. Therefore, particularly in seawater, the susceptibilities of bacterial indicators to environmental conditions should be taken into account for applications related to tracking fecal sources or waterborne pathogens. Further studies with additional environmental parameters, including the particle size distribution in river water or seawater, which can affect flocculation or sedimentation directly [29, 30], should be conducted. These studies are crucial for applying the results of recovery efficiencies or concentrations of various microbial indicators to achieve precise fecal source tracking.

## Conclusion

In conclusion, our study confirmed seasonal and spatial variations of fecal indicators using geospatial analyses in Gomso Bay, a major aquaculture area of Republic of Korea. Coliphages showed less susceptibilities of environmental conditions compared to bacterial indicators. Concentrations of both coliphages and bacterial indicators exhibited strong positive correlations. Therefore, our study preliminarily suggested that investigating fecal sources or waterborne pathogens using multiple fecal indicators combined with systemic geospatial information could be useful for tracking fecal contaminations in aquaculture products. Further longitudinal studies with predictive models that consider seasonal, spatial, and environmental variations will provide valuable information for preventing such fecal contamination in aquaculture products.

## Figures and Tables

**Table 1:** Environmental parameters for river water areas.

Parameter^a^	Sampling Period					
	Mar/2015	May/2015	Jul/2015	Sep/2015	Dec/2015	Jan/2016
Water temperature (°C)	12.1 ± 1.9	19.1 ± 1.6	22.5 ± 0.6	22.8 ± 1.5	5.3 ± 1.4	4.5 ± 0.4
pH	7.6 ± 0.4	7.1 ± 0.2	7.0 ± 0.3	7.7 ± 0.9	7.1 ± 0.5	7.6 ± 0.5
Conductivity (μS/cm)	575.9 ± 1169.0	668.8 ± 1217.3	112.5 ± 32.0	306.7 ± 332.8	173.3 ± 168.6	309.0 ± 332.0
Salinity (psu^b^)	0.4 ± 0.9	0.4 ± 0.7	0.1 ± 0.0	0.2 ± 0.2	0.1 ± 0.1	0.2 ± 0.2
Turbidity (NTU^c^)	7.0 ± 10.6	23.6 ± 34.0	15.2 ± 5.7	5.7 ± 2.7	2.6 ± 2.1	5.7 ± 2.7
Wind speed (m/s)	6.4 ± 3.3	2.8 ± 0.1	4.1 ± 1.6	1.1 ± 0.0	2.1 ± 0.6	2.4 ± 1.0
Relative humidity (%)	66.3 ± 0.1	63.6 ± 13.4	91.7 ± 1.5	80.2 ± 0.8	79.3 ± 2.8	72.2 ± 1.7
Air temperature (°C)	10.2 ± 0.1	17.4 ± 0.6	23.6 ± 0.7	19.0 ± 1.0	-0.3 ± 0.3	1.5 ± 1.1
Prep-0^d^ (mm)	0.0 ± 0.0	0.0 ± 0.0	6.8 ± 4.3	1.4 ± 0.2	0.0 ± 0.0	0.0 ± 0.0
Prep-3^e^ (mm)	34.5 ± 0.0	0.0 ± 0.0	62.5 ± 3.6	0.8 ± 0.4	0.0 ± 0.0	2.6 ± 0.6

^a^Suggested as mean ± standard deviation

^b^Practical salinity unit

^c^Nephelometric turbidity unit

^d^Cumulative precipitation on the sampling day

^e^Total cumulative precipitation from the three days preceding the sampling day

**Table 2:** Environmental parameters for seawater areas.

Parameter^a^	Sampling Period
	Jul/2015
Water temperature (°C)	24.7 ± 0.5
pH	7.7 ± 0.1
Conductivity (μS/cm)	30,757.9 ± 260.7
Salinity (psu)	30.7 ± 0.3
Turbidity (NTU)	8.0 ± 0.5
Wind speed (m/s)	1.6 ± 0.0
Relative humidity (%)	89.4 ± 0.0
Air temperature (°C)	24.7 ± 0.0
Prep-0 (mm)	0.0 ± 0.0
Prep-3 (mm)	68.1 ± 0.0

^a^Suggested as mean ± standard deviation
